# ^18^F-Labeled Peptides: The Future Is Bright

**DOI:** 10.3390/molecules191220536

**Published:** 2014-12-08

**Authors:** Susan Richter, Frank Wuest

**Affiliations:** Department of Oncology, University of Alberta, 11560 University Avenue, Edmonton, AB T6G 1Z2, Canada; E-Mail: srichter@ualberta.ca

**Keywords:** fluorine-18, peptides, labeling chemistry, microfluidic, automation, PET

## Abstract

Radiolabeled peptides have been the subject of intense research efforts for targeted diagnostic imaging and radiotherapy over the last 20 years. Peptides offer several advantages for receptor imaging and targeted radiotherapy. The low molecular weight of peptides allows for rapid clearance from the blood and non-target tissue, which results in favorable target-to-non-target ratios. Moreover, peptides usually display good tissue penetration and they are generally non-immunogenic. A major drawback is their potential low metabolic stability. The majority of currently used radiolabeled peptides for targeted molecular imaging and therapy of cancer is labeled with various radiometals like ^99m^Tc, ^68^Ga, and ^177^Lu. However, over the last decade an increasing number of ^18^F-labeled peptides have been reported. Despite of obvious advantages of ^18^F like its ease of production in large quantities at high specific activity, the low β^+^ energy (0.64 MeV) and the favorable half-life (109.8 min), ^18^F-labeling of peptides remains a special challenge. The first part of this review will provide a brief overview on chemical strategies for peptide labeling with ^18^F. A second part will discuss recent technological advances for ^18^F-labeling of peptides with special focus on microfluidic technology, automation, and kit-like preparation of ^18^F-labeled peptides.

## 1. Introduction

The deciphering of the human genome led to the identification of 483 drug targets by the entrance into the new millennium and an estimation of 5000–10,000 druggable targets on the basis of disease-relevant genes in the future [1]. Recently, Rask-Andersen *et al.* determined 475 potentially novel drug targets within the druggable human genome termed by Hopkins and Groom [1,2]. The vast majority of these drug targets (~88%) are represented by proteins. Highly specific targeting vectors comprise peptides, proteins, antibodies and antibody fragments. However, especially small peptides are ideal targeting vectors for numerous of current and future drug targets. The exquisite position of peptides among specific targeting vectors has attracted much interest from scientists of various disciplines over the last decades. In the emerging field of molecular imaging and nuclear medicine diagnosis and therapy, peptides became indispensable tools for *in vivo* visualization and monitoring of physiological and biochemical processes on the molecular and cellular level. Peptides are also attractive targeting vectors for treatment of diseases. In oncology, radiolabeled peptides have gained remarkable attention for targeted diagnostic imaging and radiotherapy. The high interest of using radiolabeled peptides for imaging and therapy stems from the overexpression of numerous specific peptide-binding receptors in various cancers and inflammatory tissues [3]. The application of peptides is furthermore justified by a manifold set of advantages. Automated solid-phase peptide synthesis (SPPS) ensures a simple and convenient synthetic access with a high degree of structural diversity to generate entire peptide libraries. Recent advances in molecular biology resulted in the development of novel techniques such as biopanning which uses phage-displayed peptide libraries for the identification of numerous molecular targets for peptide-based diagnostics and therapeutics, or to support the generation of lead structures for drug discovery. In contrast to larger targeting compounds like antibodies, peptides are characterized by a small size which allows for rapid clearance from the blood pool and non-target tissues. Good tissue penetration properties and high tumor uptake of radiolabeled peptides can lead to favorable tumor-to-background ratios as important requirement for good image quality and good cancer targeting properties in radiotherapy. Elimination from the body via excretory organs like kidneys is generally fast. Moreover, peptides are usually non-immunogenic [4].

The history of radiolabeled peptides dated back three decades when Reubi discovered an extraordinary high density of somatostatin receptors in pituitary tumors for specific targeting with radiolabeled somatostatin analogues in 1984 [5]. The first study of a radiolabeled peptide in humans was published in 1989 by Krenning *et al.* using a ^123^I-radioiodinated somatostatin analogue ([^123^I]204-090) in patients with endocrine-related carcinomas [6].

The first radiolabeled peptide approved by the US Food and Drug Administration (FDA) was ^111^In-labeled DTPA-octreotide (Octreoscan^®^) which evolved to be the gold standard for imaging of neuroendocrine tumors and remained the only regulatory approved peptide in North America and Europe for a long time. To date, most peptides for targeted molecular imaging and therapy of cancer have been labeled with radiometals.

Radiolabeling of peptides with the short-lived positron emitter fluorine-18 (^18^F) represents an attractive alternative to radiometal-based peptides. ^18^F is an ideal radionuclide for radiolabeling of small and medium-sized biomolecules like peptides. ^18^F is characterized by favorable physicochemical and nuclear properties.

This positron-emitting radionuclide exhibits high positron emission of 97%, and ^18^F can be easily produced in high yields in a small biomedical cyclotron via the ^18^O(p,n)^18^F nuclear reaction using an ^18^O-enriched H_2_O target. This allows the production of high specific activity [^18^F]fluoride in high radioactivity amounts of several hundred GBqs. Its favourable half-life of 109.8 min allows for syntheses and imaging studies over a few hours. This also allows shipping and distribution of [^18^F]fluoride and ^18^F-labeled radiopharmaceutical to facilities and hospitals without access to a cyclotron. The low positron energy of 0.64 MeV provides images with high spatial resolution due to the short maximum range in tissues (2.4 mm in water) [7]. A more accurate value for spatial resolution and tissue positron range is represented by the full width at 20% of the maximum amplitude (FW20H) of annihilation distribution and was determined to be 0.42 mm in compact bone, 0.54 mm in soft tissue, 0.58 mm in adipose tissue and 1.52 mm in lung tissue [8]. Moreover, the relatively short half-life of ^18^F causes only minor radiation doses in patients, and ^18^F-labeled peptides would also meet the needs and experience of PET clinicians with instrumentation and interpretation of PET scans as they are familiar with [^18^F]FDG (2-deoxy-2-[^18^F]fluoro-D-glucose)—the gold standard of PET imaging in oncology and other diseases [9]. However, ^18^F-labeling of peptides remains a special challenge. Direct incorporation of [^18^F]fluoride via nucleophilic aromatic substitution as one of the most prominent synthesis routes in ^18^F chemistry is usually not feasible in the case of peptides due to the required harsh reaction conditions. Other challenges include laborious and time-consuming labeling procedures and chemoselectivity aspects for the incorporation of ^18^F into peptides.

Consequently, it was not until 11 years ago that the first human PET study based on a peptide labeled with the positron emitter ^18^F has been initiated and conducted. Within this study the diagnostic performance of [^18^F]FP-Gluc-TOCA—a carbohydrated octreotide derivative labeled with the prosthetic group 4-nitrophenyl-2-^18^F-fluoropropionate—has been evaluated in comparison to Octreoscan^®^ in patients with somatostatin receptor-positive tumors [10,11].

Radiolabeled analogs of somatostatin which target somatostatin receptors became the prototype for imaging and radiotherapy of cancer with neuroendocrine origin and have been studied intensively. Somatostatin receptors belong to the class of G-protein coupled receptor family. Beyond somatostatin-based peptides to visualize somatostatin receptors, a broad range of other important peptide ligand-receptor systems have been identified for targeted molecular imaging and therapy of cancer in nuclear medicine [12]. Other prominent G-protein coupled receptors are gastrin-releasing peptide receptors (GRPRs) which can be targeted with bombesin peptide derivatives in prostate, breast, pancreatic and small-cell lung cancer or the cholecystokinin (CCK)/gastrin receptor system in colon and gastric cancers, as well as α_ν_β_3_-integrins [13]. Despite the vast number of ^18^F-labeled peptides that have been designed and preclinically evaluated over the last years, only very few ^18^F-labeled peptides (according to Li *et al.* only seven peptide-based ^18^F-radiopharmaceuticals by 2013 [14]) have been subject of clinical patient studies [10,15]. A valid explanation can be found in the challenges of ^18^F-radiosynthesis routes towards ^18^F-labeled peptide PET radiopharmaceuticals.

This review on ^18^F-labeled peptides is organized into two parts. The first part summarizes the most frequently used synthetic routes for the preparation of ^18^F-labeled peptides. The second part of the review is focused on recent technological advancements for peptide labeling with ^18^F like automation, application of microfluidic technology, and kit-like production. The review is concluded with a brief summary to highlight the potential of a bright future of ^18^F-labeled peptides for preclinical and clinical targeted molecular imaging.

## 2. General ^18^F Radiochemistry Concepts for Peptide Labeling

Two general chemical strategies are known for the radiolabeling with ^18^F using either nucleophilic substitution with no-carrier-added (n.c.a.) [^18^F]fluoride or electrophilic substitution with carrier-added (c.a.) [^18^F]fluorine gas. ^18^F-labeled peptides as radiotracers usually require high specific activity (1–10 Ci/μmol [16]) as their corresponding receptors *in vivo* are easily saturable. Moreover, peptide-binding receptors are usually expressed in quite low receptor densities *in vivo*. Thus, electrophilic radiolabeling procedures generating ^18^F-labeled compounds at low specific activity due to the presence of c.a. ^18^F-fluorine gas are not suitable for peptide labeling with ^18^F for targeted molecular imaging. Established synthesis routes towards ^18^F-labeled peptides have been focused on nucleophilic substitution approaches and are discussed in the sections below.

Conventional ^18^F-labeling procedures require harsh reaction conditions such as high temperature, organic solvents and basic conditions to introduce [^18^F]fluoride directly into target compounds. These conditions are usually not appropriate for the direct labeling of peptides with [^18^F]fluoride.

Moreover, acid side chains such as glutamic or aspartic acid in the peptide backbone may also interfere with direct nucleophilic radiofluorination reactions [4]. Hence, alternative procedures involving milder reaction conditions are needed to prepare ^18^F-labeled peptides in sufficient radiochemical yields and pharmaceutical quality.

Recently, an excellent and very detailed review on challenges and strategies for ^18^F-labeling of macromolecules was published by Kuhnast and Dollé covering three decades of research activities [17]. Three main concepts for radiolabeling of peptides with nucleophilic n.c.a. [^18^F]fluoride have been evolved over the last decades as recently compiled and illustrated by Liu *et al.* [18]. Concept 1 can be described by the activation of n.c.a. [^18^F]fluoride followed by attachment to the peptide through bioconjugation chemistry via amine and sulfhydryl groups present in the peptide backbone. The activation of [^18^F]F^−^ is achieved by generation of bifunctional labeling agents or prosthetic groups which are further reacted under mild conditions with the peptide. In return, concept 2 involves the functionalization and activation of the peptide itself and subsequent fixation of n.c.a. [^18^F]fluoride. This concept is also known as [^18^F]fluoride acceptor chemistry. Three approaches have been developed using either silicon-, boron- or aluminum-[^18^F]fluoride acceptor chemistry to radiolabel peptides within one step. This innovative methodology profits from the Lewis acid character of Si, Al, and B to form stable bonds with ^18^F. The third concept involves activation of both reaction partners - n.c.a. [^18^F]fluoride and the peptide. This dual activation concept is associated with highly prominent click chemistry methodology.

### 2.1. Concept 1: The Prosthetic Group Approach for ^18^F-Radiolabeling of Peptides

Prosthetic groups, also referred to as bifunctional labeling agents, have been used in the majority of peptide labeling approaches with ^18^F. These prosthetic groups are generated through introduction of [^18^F]fluoride into a small-molecule compound with a second functional group that allows for bioconjugation to the peptide under mild conditions. Purification from unlabeled peptide and by-products via HPLC or solid phase extraction (SPE) ensures high specific activity of the ^18^F-labeled peptide.

**Figure 1:**
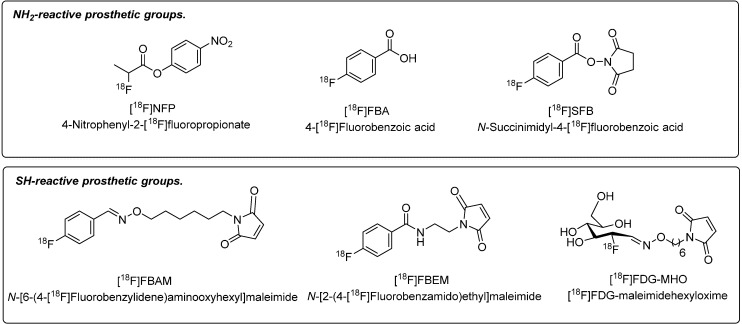
Selection of amine- and thiol-reactive prosthetic groups.

Over the years, a wide variety of different prosthetic groups have been generated that can be divided into two categories: (1) amine-reactive prosthetic groups targeting the *N*^α^-terminal amino group or the lysine *N*^ε^-amino groups of the peptide backbone via ^18^F-fluoroacylation and ^18^F-fluoroamidation reactions, and (2) thiol-reactive prosthetic groups for radiolabeling using cysteine residues and maleimides according to ^18^F-fluoroalkylation reactions. Figure 1 depicts a selection of the most frequently used prosthetic groups for peptide labeling with ^18^F.

Amine-reactive prosthetic groups 4-nitrophenyl-2-^18^F-fluoropropionate ([^18^F]NFP) and *N*-succinimidyl-4-fluorobenzoate ([^18^F]SFB) have greatly impacted the field of PET imaging using ^18^F-labeled peptides. [^18^F]NFP represents an ^18^F-acylation agent that was mostly applied to radiofluorination reactions with cyclic glycosylated pentapeptides on the basis of the RGD (Arg-Gly-Asp) sequence to give ^18^F-labeled galacto-RGD. This approach was successfully translated into the clinic for molecular imaging of α_ν_β_3_-integrins in cancer patients [19,20].

Compared to [^18^F]NFP, prosthetic group [^18^F]SFB is characterized by an aromatic [^18^F]fluorobenzoyl residue that is incorporated preferentially into peptides via conjugation to primary amine groups present in the peptide backbone. Synthesis of [^18^F]SFB was first reported in 1992 by Vaidyanathan and Zalutsky [21]. Radiosynthesis included a three-step synthesis procedure based on n.c.a. [^18^F]fluoride incorporation into the 4-formyl-*N*,*N*,*N*- trimethylanilinium-triflate followed by oxidation to form 4-[^18^F]fluorobenzoic acid and subsequent dicyclohexylcarbodiimide (DCC) activation to yield [^18^F]SFB. The total synthesis time was 100 min, and the radiochemical yield was 25%. [^18^F]SFB was first used for the radiolabeling of a monoclonal antibody F(ab')_2_. In the following years, acylation agent [^18^F]SFB became one of the most important and frequently used prosthetic groups for peptide labeling with ^18^F. The synthesis route of [^18^F]SFB was constantly improved overtime.

A microwave-assisted one-pot synthesis method involved nucleophilic radiofluorination with n.c.a. [^18^F]fluoride using *tert*-butyl (4-trimethyl-ammonium triflate)-benzoate as labeling precursor followed by acidic hydrolysis and activation of formed 4-[^18^F]fluorobenzoic acid with *O*-(*N*-succinimidyl)-*N*,*N*,*N'*,*N'*-tetramethyluronium-tetrafluoroborate (TSTU) to give [^18^F]SFB. [^18^F]SFB was purified using C18 solid-phase extraction (SPE).

Following this procedure, radiochemical yields of 44%–53% were obtained [22]. Further optimization of [^18^F]SFB synthesis included several automated synthesis procedures [23,24,25,26].

However, amine-directed prosthetic groups possess the distinctive challenge of achieving a site-selective conjugation to the peptide of interest. Radiolabeling of peptides with ^18^F on-resin represents an interesting way to introduce ^18^F via 4-[^18^F]fluorobenzoic acid ([^18^F]FBA) [27] or [^18^F]SFB [28] selectively at the *N*-terminal amine group prior to cleavage off the peptide from the resin. However, reported procedures with on-resin peptide labeling via [^18^F]SFB are time-consuming with total synthesis times over 130 min while providing only low to moderate radiochemical yields of 5%–16%. Typical overall radiochemical yields for in-solution peptide labeling with [^18^F]SFB are reported to be in the range of 30%–46% [22,29]. Recently, radiochemical yields for solid-phase peptide conjugation using 4-[^18^F]fluorobenzoic acid could be increased to 35%–64% in dependency of solid support and cleavage conditions [30]. Also, ^18^F-fluoropropionic acid ([^18^F]FPA) has been employed as alternative to 4-[^18^F]fluorobenzoic acid to radiolabel peptides on solid support since ^18^F-FPA may not alter size and lipophilicity as much as the aromatic ^18^F-FBA [31,32]. Synthesis times were above 171 min generating ^18^F-FPA-peptides conjugated selectively to either the *N*-terminus or the Lys-side chain in radiochemical yields of 3%–10%.

Also, various prosthetic groups on the basis of thiol-reactive ^18^F-labeled maleimides (Figure 1), have been developed to address the challenge chemoselectivity since maleimides undergo site-specific reactions with sulfhydryl groups according to a Michael addition. Hence, cysteine-containing peptides—naturally occurring or cysteine residue-modified—are suitable for this radiolabeling approach. Prominent examples of sulfhydryl-reactive maleimide-based prosthetic groups are *N*-[6-(4-[^18^F]fluoro-benzylidene)aminooxyhexyl]maleimide ([^18^F]FBAM) [33,34], *N*-[2-(4-[^18^F]fluorobenz-amido)ethyl]maleimide ([^18^F]FBEM) [35] or [^18^F]FDG-maleimidehexyloxime ([^18^F]FDG-MHO) [36].

### 2.2. Concept 2: ^18^F-Radiolabeling of Peptides via [^18^F]Fluoride Acceptor Chemistry

The [^18^F]fluoride acceptor chemistry represents a direct and elegant labeling method for peptides with fluorine-18 exploiting the formation of stable Si-^18^F, B-^18^F or Al-^18^F bonds. The radiolabeling proceeds through an isotopic exchange reaction of ^19^F with ^18^F.

The strong nature of the Si-F bond prompted the investigation of [^18^F]fluoride substitution at organosilicon synthons (SiFA, silicon-fluoride-acceptor) and modified peptides [37]. Positive attributes like little precursor amount (μg range) and high specific activity illustrate the advantages of this reaction.

However, a challenge is the hydrolytic stability *in vivo* of ^18^F-organosilicon compounds depending on the substitution pattern of the silicon moiety [38]. Hydrolytic degradation can be significantly reduced by the introduction of bulky substituents like *tert*-butyl groups to the silicon moiety.

However, bulky substituents like *tert*-butyl groups drastically increase the lipophilicity of the peptide and results in high intestine, liver and gall bladder uptake as demonstrated by Hoehne *et al.* using several ^18^F-labeled organosilico-bombesin derivatives [39]. The introduction of hydrophilic spacer like PEG and carbohydrates into ^18^F-SiFA-tagged bombesin and RGD derivatives led partially to a compensation of the lipophilic nature, and therefore reduced logD values as demonstrated for ^18^F-labeled SiFA-LysMe3-γ-carboxy-d-Glu-RGD peptide [40]. Recently, the development of the ^18^F-SiFA approach including its application for peptide radiolabeling has extensively been reviewed [41].

The Perrin group studies boron-^18^F acceptor chemistry as an alternative approach which led to the development of [^18^F]aryltrifluoroborate ([^18^F]ArBF_3_) bioconjugates. In 2011, they reported the radiolabeling of a boronic acid ester-modified marimastat peptide for molecular imaging of matrix metalloproteinases in breast cancer [42]. Isolated radiochemical yields were only 2%–4%. A special challenge of this methodology is the need to work in low reaction volumes of about 1.5 μL. Recently, Perrin and colleagues improved the initial reaction conditions by replacing bulky aryltrifluoroborates with alkylammoniomethyltrifluoroborate (AMBF_3_) groups. Octreotate decorated with AMBF_3_ was subjected to an ^18^F-^19^F isotopic exchange reaction using n.c.a. [^18^F]fluoride to yield the corresponding ^18^F-labeled peptide within 25 min including C18-SPE purification. Radiochemical yields were in the range of 20%–25% and specific activity was determined to be 111 GBq/μmol [43]. The repertoire of high specific activity ^18^F-labeled peptides based on ^18^F-B acceptor chemistry could successfully be extended to trimeric RGD peptides and dual-mode fluorescent-dimeric RGD [44].

The Al-^18^F acceptor chemistry method combines the convenient chelator-based radiolabeling using minute amounts of peptide (nmol range) with favorable physicochemical characteristics of ^18^F. McBride *et al.* pioneered the radiolabeling of a hapten peptide with ^18^F according to this method. The reported uncorrected radiochemical yield was 5%–20%. An aqueous AlCl_3_ solution in sodium acetate buffer (pH 4) was mixed with cartridge-purified [^18^F]fluoride to give Al-^18^F complex which was reacted with the NOTA-functionalized peptide for 15 min without the need of further purification [45]. Conventional time-consuming azeotropic drying of [^18^F]fluoride was not necessary. The Al-^18^F complex is stable and no deradiofluorination was observed *in vivo*. The portfolio of Al-^18^F-labeled peptides reported in the literature is mostly based on octreotide [46], a dimeric cyclic RGD peptide (E[c(RGDyK)]_2_) [47,48] and bombesin [49,50].

**Figure 2:**
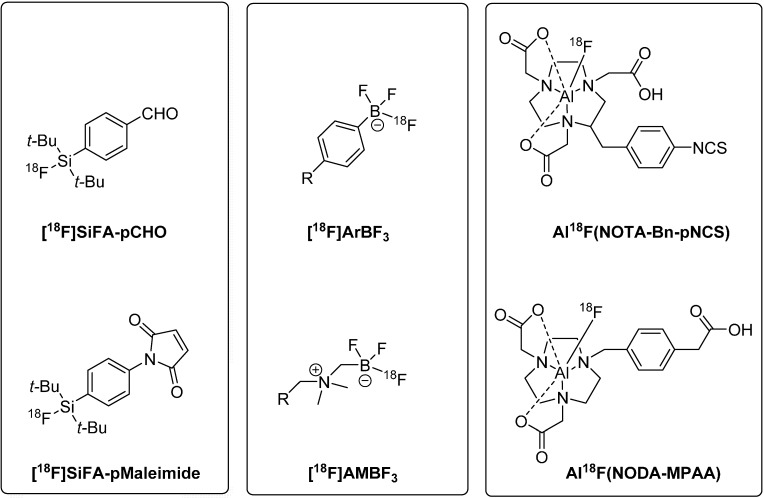
Prominent Si-^18^F, B-^18^F, and Al-^18^F building blocks used for direct labeling of peptides with F-18 (Si-^18^F building blocks: aldehyde (**top**) and maleimide (**bottom**) derivative of the di-*tert*-butyl-modified silicon fluoride acceptor species; B-^18^F building blocks:aryltrifluoroborate (**top**) and alkylammoniomethyltrifluoroborate (**bottom**); Al-^18^F building blocks: S-2-(4-isothiocyanatobenzyl)-1,4,7-triazacyclononane-1,4,7-triacetic acid species (**top**) and 1,4,7-triazacyclononane-1,4-diacetic acid-methylphenylacetic acid derivative (**bottom**)).

Figure 2 gives an overview on a selection of prominent Si-^18^F, B-^18^F, and Al-^18^F building blocks for peptide radiolabeling via [^18^F]fluoride acceptor chemistry.

### 2.3. Concept 3: Click Chemistry for Radiolabeling of Peptides with Fluorine-18

Click chemistry is defined as a bioorthogonal, high-yielding, fast and chemo- and stereoselective reaction. Over the last decade, click chemistry has become a powerful and versatile synthesis approach in radiopharmaceutical chemistry [51,52,53]. The term click chemistry has been shaped by Sharpless, and it initially referred to the 1,3-dipolar Huisgen cycloaddition which is characterized by the formation of a triazole moiety through the copper(I)-catalyzed reaction of an alkyne with an azide [54]. Historically, the copper(I) catalyst was generated *in situ* from Cu(II) sulfate. More recently, copper(I) salts such as CuI or CuBr have been used directly [55]. The click chemistry methodology can also be considered as prosthetic group approach. Due to its advantageous reaction condition involving fast, chemo- and regioselective reactions in aqueous media, click chemistry has also been exploited for ^18^F-radiolabeling of peptides.

Marik and Sutcliffe pioneered this reaction in the field of radiopharmaceutical chemistry. They performed a Cu(I)-mediated click chemistry between azidopropionic acid-decorated model peptides radiolabeled with various ω-[^18^F]fluoroalkynes. The reactions proceeded within 10 min in excellent radiochemical yields of 55%–99% [56].

Ramenda *et al.* tackled and solved the problem of the potentially low *in vivo* stability connected with aliphatic ^18^F-labeled alkynes by the introduction of an aromatic ^18^F-labeled alkyne building block [57]. Therein, [^18^F]fluoro-*N*-(prop-2-ynyl)benzamide was used for effective radiolabeling of an azido-pentanoic acid-linked neurotensin(8-13) peptide.

The reaction proceeded in high radiochemical yields of 70% within 20 min while using only minor peptide amounts of 0.5–1 mg. Various ^18^F-labeled azides have also been developed for radiolabeling of respective alkyne-functionalized peptides as an alternative approach. Glaser *et al.* used 2-[^18^F]fluoroethylazide for click chemistry with alkyne-functionalized pentapeptide. The obtained radiochemical yield was 92% within 15 min at room temperature [58]. Thonon *et al.* developed aromatic building block 1-(azidomethyl)-4-[^18^F]fluorobenzene which is more resistant towards *in vivo* radiodefluorination. Radiolabeling of alkyne-modified [Leu^5^]enkephalin neuropeptide afforded the ^18^F-labeled triazole-peptide derivative at room temperature in 95% radiochemical yield within 15 min [59]. The first *in vivo* imaging approach utilizing a ^18^F-labeled peptide prepared via click chemistry was reported by Li *et al.*^18^F-PEGylated alkyne-labeled RGD peptide dimer demonstrated better *in vivo* stability due to the multimeric nature of the peptide which also led to more favorable tumor targeting efficiency [60]. A click-based ^18^F-carbohydration two-step synthesis procedure (including purification) for ^18^F-Galacto-RGD was reported by Maschauer *et al.* [61]. [^18^F]Fluoride acceptor chemistry was also combined with Cu(I)-mediated 1,3-dipolar cycloaddition, where an alkyne-modified ^18^F-aryltrifluoroborate anion was reacted with only microgram quantities of an azido-bombesin antagonist peptide [62].

Recently, inverse-electron demand Diels Alder reactions of electron-deficient tetrazines with ring-strained *trans*-cyclooctenes or norbornenes were explored as copper-free click chemistry approaches in radiopharmaceutical chemistry. These powerful reactions are very useful for innovative *in vivo* pretargeting approaches. Several strain-promoted reactions applying azide-decorated peptides (octreotate [63], α_ν_β_6_ integrin-targeting peptide A20FMDV2 [64]) and ^18^F-labeled cyclooctyne species were reported as versatile novel bioconjugation tools. Click chemistry functionalities are interchangeable as demonstrated for the reaction of an ^18^F-labeled aliphatic azide with a cyclooctene-modified bombesin peptide. The obtained radiochemical yield was of 37% [65]. The very fast reaction of tetrazines with ^18^F-labeled *trans*-cyclooctene species led to the introduction of various tetrazine-functionalized peptides.

Very small amounts of a tetrazine-functionalized RGD peptide (30μg) were reacted with ^18^F-labeled *trans*-cyclooctene by Selvaraj *et al.* to afford radiolabeled peptide in over 90% radiochemical yields within 5 min. PET imaging in U87MG-bearing mice revealed prominent tumor uptake of the copper-free click chemistry-generated ^18^F-labeled RGD derivative [66]. Among others, our group pioneered the application of copper-free click chemistry for the synthesis of a stabilized bombesin peptide functionalized with a tetrazine-moiety [67]. Reaction of tetrazine-functionalized bombesin with [^18^F]SFB-derived norbornene derivative gave dihydropyradazine-containing bombesin derivative as an alternative strain-promoted click chemistry methodology. The diversity of click chemistry reactions combined with its simple, fast and chemoselective nature equips (radio)chemists with a versatile chemistry tool for the production of ^18^F-labeled peptides with high potential for translation into clinical practice.

Furthermore, click chemistry according to concept 3 also includes activation of peptides via aminooxy- or hydrazine-modification. Functionalized peptides can subsequently be radiolabeled with 4-[^18^F]fluorobenzaldehyde ([^18^F]FBA) or [^18^F]FDG to form corresponding oximes or hydrazones. Click chemistry-related oxime and hydrazone formation represents another innovative tool for chemoselective bioconjugation reactions. These beneficial one-step, high yielding syntheses (greater 60%) require only small amounts of peptide in the sub-milligram scale.

Recent literature describes a broad range of clinically-relevant peptides such as [^18^F]FBA-labeled minigastrin, RGD and octreotide derivatives [68,69], as well as [^18^F]FDG-modified neurotensin(8–13) [70] and cycloRGD [71]. Additionally, the advantage of using [^18^F]FDG for peptide labeling is twofold. Beyond the straightforward one-step oxime-forming reaction, the peptide is exposed to a carbohydration reaction which subsequently can improve radiopharmacokinetic profile *in vivo*.

## 3. Recent Technology Advances in Peptide Labeling with ^18^F

### 3.1. Automated Synthesis of ^18^F-Labeled Peptides

Transition of ^18^F-labeled peptides into clinics requires a radiosynthesis set-up which allows safe and reliable handling of large amounts of radioactivity to minimize radiation exposure. Automation and routine production require preferentially simple synthesis protocols with only a few reaction steps to yield the desired ^18^F-labeled radiotracer. In general, the synthesis, purification, analysis and formulation of the radiopharmaceutical should not exceed two half-lives of the used radionuclide. This also fully applied to the automated radiosynthesis of ^18^F-labeled peptides for clinical applications. Recent progress led to the development of fully-automated, remotely-controlled radiosyntheses of ^18^F-labeled peptides.

Introduction of the first automated radiosynthesis of prosthetic group [^18^F]SFB in a remotely-controlled synthesis module was reported by Maeding *et al.* in 2005, yielding [^18^F]SFB in 34%–38% radiochemical yield within a total synthesis time of 68 min [23]. Optimization attempts involving precursor amounts and C18 SPE purification step further improved the automated [^18^F]SFB synthesis. Currently, our laboratory prepares [^18^F]SFB on a routine basis in high radiochemical yields of 71% ± 20% within 63 min in a GE TRACERlabFx synthesis unit. [^18^F]SFB is purified via SPE, and the obtained specific activity is higher 40 GBq/μmol. Efficient radiolabeling of peptides with module-prepared [^18^F]SFB was recently demonstrated by the radiosynthesis of [^18^F]fluorobenzoyl-modified bombesin derivative [^18^F]FBz-Ava-QWAV-Sar-H-FA01010-Tle-NH_2_ [29]. The peptide could be prepared in patient relevant activity amounts of ~200 MBq. An alternate automated [^18^F]SFB synthesis approach based on the ethyl (4-trimethylammonium triflate)-benzoate as labeling precursor was reported by Tang *et al.* in 2008 [24]. Automated [^18^F]SFB synthesis was performed on a new generation FASTLab^TM^ GE synthesis module [25].

**Figure 3:**
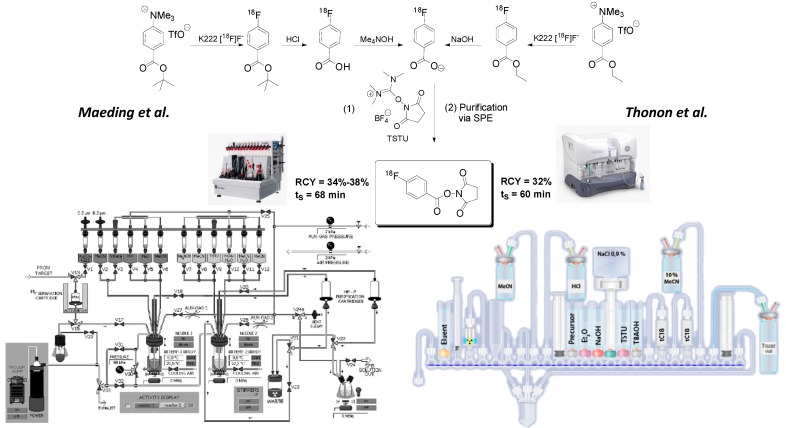
Module-assisted preparation of [^18^F]SFB according to Maeding *et al.* [23] (**left**) in a GE TRACERlabFx module using the acidic hydrolysis of ^18^F-labeled *tert*-butylester intermediate and according to Thonon *et al.* [25] (**right**) in a GE FASTlab^TM^ module via basic hydrolysis of intermediate 4-[^18^F]fluorobenzoylethyl ester.

Figure 3 depicts radiosynthesis of [^18^F]SFB in commercially available synthesis units according to the methods described by Maeding *et al.* and Thonon *et al.* Additionally, the [^18^F]SFB labeling of peptide PRGD2 (an RGD derivative) was included into the automated synthesis method by Thonon *et al*.

The presented (semi)automated synthesis approach afforded up to 4 GBq of HPLC-purified ^18^F-fluorobenzoylated PRGD2 peptide starting from 70 GBq of [^18^F]fluoride within a total synthesis time of 130 min. The decay-corrected radiochemical yield was 13%.

A short conference abstract by Marik *et al.* is indicating the development and presence of an automated on-resin synthesis for ^18^F-labeled peptides [26]. Peptides on solid support were radiolabeled with [^18^F]FBA and [^18^F]FPA and cleaved in a second step using a programmable automatic syringe pump equipped with a 8-port head simulating a continuous flow synthesizer. Reported radiochemical yields were comparable to the manually performed ^18^F-labelling via SPPS with crude yields above 90%.

Alternatively, fully remotely-controlled and highly robust radiosynthesis methods are available for sulfhydryl group-reactive prosthetic groups. *N*-[6-(4-[^18^F]fluorobenzylidene)aminooxy-hexyl]maleimide (^18^F-FBAM) can be prepared on a GE TRACERlabFx module in radiochemical yields of 29% within 40 min including SPE purification [33,34]. Prosthetic group *N*-[2-(4-[^18^F]fluorobenzamido)ethyl]maleimide (^18^F-FBEM) was prepared on an Eckert and Ziegler synthesis module in 13% radiochemical yields within 95 min including HPLC purification [35].

In summary, several automated radiosyntheses of prominent prosthetic groups have successfully been developed. However, a fully automated procedure for the radiosynthesis of clinically relevant ^18^F-labeled peptides without manual intervention is still pending.

A recent report by Ackermann *et al.* directs the way towards this goal as a fully automated synthesis of [^18^F]FBEM-labeled model peptide glutathione could be achieved in an iPHASE FlexLab synthesis module [72].

### 3.2. Application of Microfluidic Technology for ^18^F-Labeling of Peptides

A highly flexible and versatile approach addressing aspects of chemoselectivity, required amounts of peptide precursor and synthesis time represents microfluidic technology. Application of microfluidic devices allows for rapid synthesis of radiolabeled peptides in high radiochemical yields by using only minute amounts of peptide precursor making this technology a promising tool for the synthesis of ^18^F-labeled peptides as PET radiotracers for molecular imaging [73]. The fully-automated control of the microfluidic device supports safe handling of radioactivity.

This novel technology is particularly advantageous for the radiolabeling of highly complex peptides as it enables purer product formation and chemoselectivity. A prominent example is cell-penetrating phosphopeptide containing several lysine and arginine residues which gave a highly complex and difficult to purify reaction mixture when radiolabeling was performed with [^18^F]SFB using conventional labeling conditions in a small reaction vial.

On the other hand, performance of the labeling reaction in a microfluidic reactor predominantly led to the reaction of [^18^F]SFB on the *N*-terminus of the peptide which resulted in much cleaner product formation [74]. Radiochemical yields were increased to 26% compared to 2% via the conventional radiolabeling procedure. Reaction times were reduced to 12 min, and peptide precursor amounts could also significantly be reduced. The improved chemoselectivity favoring acylation reaction of [^18^F]SFB on the *N*-terminal end of the peptide can possibly be explained by the masking of the arginine and lysine residues by the surface of the capillary-like microreactor. Moreover, the capillary design of the microreactor provides an enlarged specific surfaces which leads to a more efficient transfer and exchange of material and heat in course of the reaction [75]. Recently, microfluidic methodology was applied to the radiosynthesis of a clinically relevant octreotide TATE derivative [^18^F]FDG-TATE [76].

**Figure 4:**
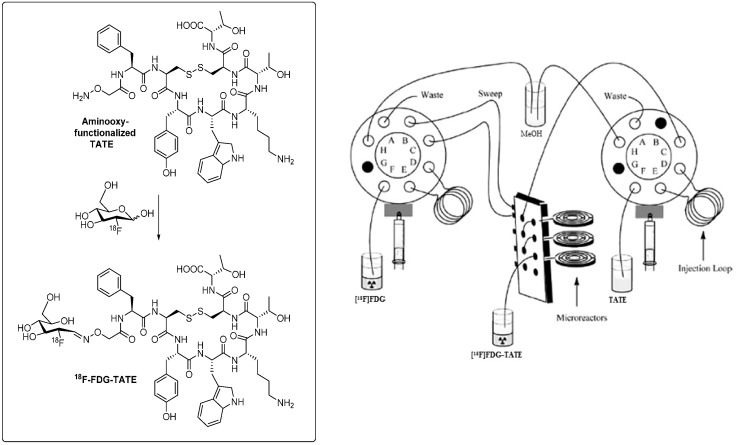
Radiosynthesis of ^18^F-labeled octreotide derivative [^18^F]FDG-TATE in a microfluidic synthesis apparatus. (Adapted and modified from: Bouvet and Wuest, 2013 [76].)

The aminooxy-functionalized TATE derivative was labeled with [^18^F]FDG in high radiochemical yields of greater than 82%. Figure 4 depicts an outline of the microfluidic-based synthesis set-up to prepare [^18^F]FDG-TATE. Radioactivity level as relevant for the preparation of patient doses could also be applied. This result demonstrates principle feasibility to use microfluidic technology for the synthesis of ^18^F-labeled peptides for clinical applications.

### 3.3. Kit-Like Preparation of ^18^F-Labeled Peptides

“A next generation” of ^18^F labeling methodology for peptides has recently been described by using a “kit-like” labeling protocol according to the well-established kit preparation of ^99m^Tc- and ^188^Re-labeled radiopharmaceuticals. This procedure allows for highly efficient and reproducible radiolabeling reactions as required for clinical applications. Current efforts are directed towards the development of kit-like procedures exploiting ^18^F-SiFA and ^18^F-ArBF_3_ chemistry, as well as consideration of a recently developed simple and fast ^18^F drying method via anion exchange cartridges [77,78,79]. To date, only peptide labeling according to Al-^18^F chemistry was successfully used in a true kit-like preparation. The formation of the Al^18^F complex occurs in aqueous solution eliminating time-consuming drying steps and permits the use of USP grade [^18^F]fluoride in saline [80]. Critical reaction parameters are pH (optimal: pH 4) and temperature (~100 °C). *In vitro* and *in vivo* stable Al^18^F bonds can be generated by complexation with NOTA as chelating agent [81]. The choice of the chelating agent is an important parameter. Another promising ligand for labeling with the (Al-^18^F)^2+^ species is NODA (1,4,7-triazacyclononane-1,4-diacetate) lacking an acetic acid in comparison to NOTA.

Shetty *et al.* reported consistently higher labeling efficiency for NODA compared to NOTA suggesting an interence of the third carboxylic group with the binding of ^18^F-fluoride to aluminium. Also various NODA derivatives with carbonyl functions at least 3–4 carbons distant from the chelator, such as NODA-MPAA (methylphenylacetic acid), can be radiolabeled in high yields (>78%) as opposed to NODA derivatives having a carbonyl group adjacent to the chelator ring. Subsequent formation of 5- or 6-membered rings with NODA reduce the labeling yield [80,82].

**Figure 5:**
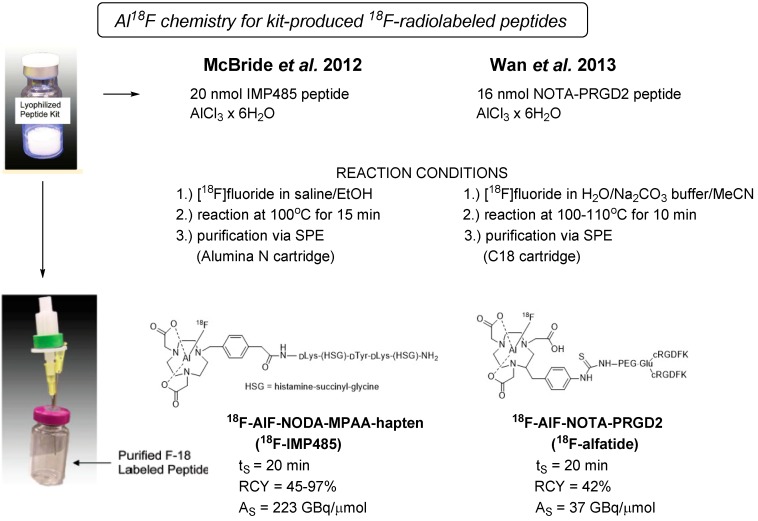
Lyophilized kit procedure using the Al^18^F chelator chemistry for ^18^F radiolabeling of hapten peptide IMP485 (Mc Bride *et al.* [83]) in comparison to dimeric RGD derivative alfatide (Wan *et al.* [84]). (Pictures of the kit vials were adapted from [83].)

Figure 5 gives an overview on the kit-like preparation of ^18^F-labeled peptides as reported in the literature by McBride *et al.* and Wan *et al.* [83,84]. The scheme illustrates the simplicity of the ^18^F-Al chelating chemistry approach avoiding HPLC purification. McBride and coworkers successfully developed a versatile and highly reproducible kit-labeling protocol of peptides as exemplified with ^18^F-labeled NODA-MPAA and NOTA-modified hapten peptides. The kit-prepared ^18^F-labeled dimeric RGD peptide (^18^F-alfatide) reported by Wan *et al.* was introduced into the clinic, and its feasibility was demonstrated in lung cancer patients with squamous or adenomatous carcinoma. The simple handling of lyophilized kits used for radiolabeling of PRGD2 peptide affords ready-to-use ^18^F-AlF-NOTA-PRGD2 (^18^F-alfatide) in 42% radiochemical yield within 20 min including C18-SPE purification [84].

More recently, attempts were made to apply click labeling with ^18^F-ArBF_3_- into an easy-to-use “kit-like” procedure using cycloRGD peptides [78]. The overall synthesis time was 2 h, and the non-decay-corrected radiochemical yield was only 4%. The obtained low specific activity was due to the use of carrier-added [^18^F]KHF_2_. Further optimization is needed to apply this protocol to ^18^F-labeled peptides for subsequent clinical applications.

## 4. Challenges and Trends in Peptide Receptor-Targeted Molecular Imaging

Beyond highly promising recent chemical developments towards the synthesis of clinically relevant ^18^F-labeled peptides such as automation and kit-like preparation, additional pharmacological aspects for the use of ^18^F-labeled peptides in nuclear medicine have to be considered.

Recently, two major “breakthroughs” have been published which have the potential to revolutionize the clinical application of ^18^F-labeled peptides in the future.

One of them involves the peptides interaction with its receptor. It was widely believed that peptides that act as receptor agonists are superior for optimal tumor targeting with high tumor uptake.

Studies using peptide receptor antagonists ^177^Lu-DOTA-sst2 demonstrated that more binding sites can be targeted merely by ligand-receptor interaction instead of subsequent internalization as typical for receptor agonists. The work of Cescato *et al.* pinpoints this change in paradigm for radiolabeled peptides [85]. Furthermore, the absence of internalization and induction of second messenger responses in the case of peptide receptor-based radiotherapy avoids pharmacological side effects as valid criteria. Another breakthrough is related to the recently introduced “to protect and serve” concept by Nock *et al.* [86]. This concept deals with the improvement of metabolic stability *in vivo* as a key element for successful tumor targeting with peptides in cancer. It involves co-administration of protease inhibitors like phosphoramidon with a range of unstabilized radiometal-labeled peptides (somatostatin, bombesin and minigastrin) instead of using other tedious classical synthetic stabilization methods for peptides, including multimerization. It was shown that the residency time of intact radiopeptides in the circulation was significantly extended when a protease inhibitor was co-injected. This resulted in significantly enhanced tumor uptake of radiolabeled peptide in various mouse xenografts.

This concept was first reported by Bergmann *et al.* when the authors observed an enhanced half-life of a stabilized ^18^F-labeled neurotensin derivatives in arterial rat blood *in vivo* when co-injected with protease inhibitors thiorphan and bacitracin [87]. Scope and limitations towards a translation of this concept into clinical practice needs to be investigated in the future since administration of pharmacological doses of protease inhibitors in patients have the potential to cause severe toxicological side effects.

## 5. Summary and Conclusions

In recent years, numerous radiolabeled peptides for diagnostic and therapeutic application in nuclear medicine have been designed and synthesized. This trend has stimulated the development of a multitude of innovative synthetic routes and technology advancements towards the preparation of ^18^F-labeled peptides. The majority of these procedures involve the incorporation of ^18^F via prosthetic groups. Click chemistry as versatile synthesis tool for bioconjugation enjoys increasing popularity in the world of radiopharmaceutical chemistry. ^18^F-labeling of peptides using novel techniques such as microfluidic technology offer several advantages to conventional radiolabeling methods resulting in shorter reaction times, more efficient radiochemistry, improved chemoselectivity, and more economical use of starting material. Developments towards efficient fully-automated radiosyntheses of ^18^F-labeled peptides will further stimulate and inspire the field in the future since it represents a highly promising synthesis pathway to translate more ^18^F-labeled peptides into the clinic.

Despite the successful and efficient automation of several ^18^F-prosthetic groups for peptide labeling in patient-relevant doses, a fully remotely-controlled synthesis procedure to yield ^18^F-labeled peptides starting from n.c.a. [^18^F]fluoride still awaits its development. Thonon *et al.* were the first to produce up to 4 GBq of a ^18^F-labeled RGD peptide derivative on a GE FASTlab synthesis unit using a semi-automated procedure which still required manual addition of the peptide precursor into the system [25]. A fully automated synthesis of model peptide glutathione with [^18^F]FBEM was reported in 2014 [26]. This report raises confidence that the first fully automated synthesis of a ^18^F-labeled peptide with clinical potential is only a minor step ahead. An appealing alternative to preparation ^18^F-labeled peptides via prosthetic group conjugation is [^18^F]fluoride acceptor chemistry. It benefits from the replacement of time-consuming, low yielding multi-step synthesis procedures. Moreover it is applicable to simple one-step lyophilized kit-like preparation of ^18^F-labeled peptides. Successful implementation of this approach was reported very recently using the Al-^18^F chemistry by McBride *et al.* and Wan *et al.* [83,84]. Hence, the Al-^18^F method symbolizes a highlight procedure for the generation of ^18^F-labeled peptides in a clinical environment to date. More kit-like approaches using silicon- and boron-[^18^F]fluoride chemistry to produce ^18^F-labeled peptides will probably follow soon.

Recent innovative chemical and technology advancements combined with recent important findings in radiopeptide pharmacology will provide an efficient and elegant platform for the routine preparation and application of various ^18^F-labeled peptides in clinical research and practice in the near future. These developments will lead ^18^F-labeled peptides into a bright future.
